# Mayaro Virus Replication Restriction and Induction of Muscular Inflammation in Mice Are Dependent on Age, Type-I Interferon Response, and Adaptive Immunity

**DOI:** 10.3389/fmicb.2019.02246

**Published:** 2019-10-01

**Authors:** Camila Menezes Figueiredo, Romulo Leão da Silva Neris, Daniel Gavino-Leopoldino, Mariana Oliveira Lopes da Silva, Juliana Silva Almeida, Julio Souza dos-Santos, Claudia Pinto Figueiredo, Maria Bellio, Marcelo Torres Bozza, Iranaia Assunção-Miranda

**Affiliations:** ^1^Instituto de Microbiologia Paulo de Goes, Universidade Federal do Rio de Janeiro, Rio de Janeiro, Brazil; ^2^Faculdade de Farmácia, Universidade Federal do Rio de Janeiro, Rio de Janeiro, Brazil

**Keywords:** mayaro virus, pathogenesis, replication restriction, type-I interferon response, adaptive immunity, muscle inflammation

## Abstract

Mayaro virus (MAYV) is an emergent arbovirus first described in forest regions of the American continent, with recent and increasing notification of urban area circulation. Similar to Chikungunya (CHIKV) and other arthritogenic Alphavirus, MAYV-induced disease shows a high prevalence of persistent arthralgia, and myalgia. Despite this, knowledge regarding pathogenesis and characteristics of host immune response of MAYV infections are still limited. Here, using different ages of wild-type (WT), adult Type I Interferon receptor deficient (IFNAR^–/–^), and adult recombination activation gene-1 deficient (RAG^–/–^) mice, we have investigated the dependence of age, innate and adaptive immunity for the control of MAYV replication, tissue damage, and inflammation in mice. We have found that MAYV induces clinical signal and replicates in young WT mice, which gain the ability to restrict MAYV replication with aging. In addition, we observed that mice age and type I interferon response are related to restriction of MAYV infection and muscular inflammation in mice. Moreover, MAYV continues to replicate persistently in RAG^–/–^ mice, being detected at blood and tissues 40 days post infection, indicating that adaptive immunity is essential to MAYV clearance. Despite chronic replication, infected adult RAG^–/–^ mice did not develop an apparent signal of muscle damage in early and late infection. On the other hand, MAYV infection in young WT and adult IFNAR-/- mice triggers an increase in the expression of pro-inflammatory mediators, such as TNF, IL-6, KC, IL-1β, MCP-1, and RANTES, in muscle tissue, and decreases TGF-β expression, that were not significantly modulated in adult WT and RAG^–/–^ mice. Taken together, our data demonstrated that age, innate and adaptive immunity are important to restrict MAYV replication and that adaptive immunity is also involved in MAYV-induced tissue damage. These results contribute to the comprehension of MAYV pathogenesis, and describe translational mice models for further studies of MAYV infection, vaccine tests, and therapeutic strategies against this virus.

## Introduction

Mayaro virus (MAYV) is an *Alphavirus* from the Togaviridae family, transmitted to humans mainly by the bites of *Haemagogus* mosquitoes (Esposito and Fonseca, 2017). MAYV was first isolated in 1954 from a febrile case in Trinidad and Tobago and maintained until the present day on restricted circulation in Central and South American forest regions on sporadic outbreaks (Azevedo et al., 2009; Mourao et al., 2012; Auguste et al., 2015). However, recent studies indicate that the number of reported MAYV cases could be underestimated, warning for the risk of emergence, dispersion to new areas, and for the potential establishment of an urban epidemic cycle (Long et al., 2011; Mackay and Arden, 2016; Brunini et al., 2017; da Costa et al., 2017; Esposito and Fonseca, 2017; Mavian et al., 2017). Even in face of such risks, information regarding MAYV infection and mainly the molecular mechanism of pathogenesis is still very limited.

Due to the profile of clinical manifestations, MAYV is grouped with the arthritogenic Alphavirus such as Chikungunya (CHIKV) and Ross River (RRV). MAYV infection promotes a febrile condition that presents a set of unspecific signs and symptoms, such as rash, headache, and ocular pain, which facilitates its misdiagnosis as other arboviroses such as dengue fever (Tesh et al., 1999; Mourao et al., 2012; Zuchi et al., 2014; Smith et al., 2018). Moreover, MAYV infected patients present a high incidence of articular and muscular pain (Mourao et al., 2012; da Costa et al., 2017), reaching about 50 and 77% of patients in some outbreaks, respectively (Tesh et al., 1999). In addition, it has also been reported that myalgia and articular symptoms of MAYV infections could persist for months, revealing a common feature to arthritogenic alphavirus-induced disease (Taylor et al., 2005; Halsey et al., 2013; Theilacker et al., 2013; Slegers et al., 2014).

High activation of immune response has been described in CHIKV and RRV-infected patients presenting acute and persistent symptoms (Chow et al., 2011; Tappe et al., 2017). Analyses of muscle biopsies of CHIKV-infected patients with severe polyarthralgia and myalgia showed that symptoms persistence was associated with long-term cellular infiltrate at articular and muscle tissue (Ozden et al., 2007). However, the characteristics of the immune response induced by MAYV, the mechanisms of resolution of the infection or symptoms persistence are largely unknown. The one-year longitudinal study of Santiago et al., 2015 demonstrated that MAYV-infected patients also present prolonged immune response, with high concentrations of pro-inflammatory mediators in their serum (Santiago et al., 2015). They found lower amounts of GM-CSF, IL-5, and IL-10 in MAYV-infected patients when compared to CHIKV patients, which indicates differences in the profile of the induced immune response. Consistent with this, a difference in cytokine expression between MAYV and CHIKV infection in human U937 cell lineage (Danillo Lucas Alves and Benedito Antonio Lopes da, 2018) was observed. However, contrastingly from what was observed in patients, MAYV infected U937 cells display a more anti-inflammatory profile of immune activation. Despite the divergence, this data reinforces the necessity of further studies that evaluate cellular and molecular aspects of MAYV infection.

The muscle and joint inflammation during CHIKV and RRV have been evaluated in immunocompetent and immunodeficient mice, as well as in non-human primates (Lidbury et al., 2008; Labadie et al., 2010; Ganesan et al., 2017). It was demonstrated that inflammatory monocyte infiltrates trigger tissue damage, contributing to the severity of the disease (Haist et al., 2017). However, there are few studies evaluating replication and the role of immune activation in MAYV-infected mice (Taylor et al., 2015; Santos et al., 2019). Here we investigated age, innate and adaptive immunity dependence for MAYV replication, and induction of tissue damage. We observed that MAYV replication and dissemination in wild-type (WT) SV129 mice is determined by aging and is controlled by innate immunity. We also demonstrated that adaptive immunity is essential to MAYV clearance. Finally, MAYV infection promotes an inflammatory process in the muscle of young WT mice, with high amounts of pro-inflammatory mediators. Taken together, our data contribute to the comprehension of MAYV pathogenesis and for further studies of MAYV infection and for testing therapeutic strategies against this arbovirus.

## Materials and Methods

### Virus

Mayaro virus (ATCC VR 66, strain TR 4675 – isolated from the serum of a patient, 1954), was propagated at successive passages in BHK-21 (ATCC-CCL-10), and cultured in α-MEM (alfa-Minimum Essential Medium – Invitrogen). BHK-21 cells were infected in a multiplicity of infection (MOI) of 0.1 and 30 h post infection (hpi) culture medium was collected and centrifuged at 2,000 × *g* for 10 min to remove cell debris, aliquoted and stored at −80^o^C. Viral titer of the stock was determined by plaque assay (described in section “Virus Quantification”).

### Animals and Infection

The experiments were performed using young WT SV129 mice (6, 11, and 21 days after birth); as well as adult (8 weeks old) WT SV129, type I interferon receptor deficient mice (IFNAR^–/–^), WT C57BL/6, and recombination activation gene RAG-1 deficient C57BL/6 mice (RAG^–/–^). Mice were subcutaneously inoculated in the left footpad with 10^6^ pfu of MAYV, using a final volume of 20 μL. Only for the infection of IFNAR^–/–^ mice the inoculation was that of 10^5^pfu of MAYV. The same volume of virus-free BHK-21 medium (Mock) was used as the control. Each experimental group was housed individually in polypropylene cages with free access to chow and water. Young mice were housed with the uninfected mother during all the experiment.

Mice were weighed daily and clinical signals were scored. The area of hind limb foot edema in IFNAR^–/–^ animals was determined from the width-height measurements of the metatarsal region using a digital caliper. Tissue samples were collected at 2 and 4 days post infection and stored at −80^o^C until processed or fixed in 4% formaldehyde.

### Ethics Statement

All experimental procedures performed were in accordance with protocol and standards established by the National Council for Control of Animal Experimentation (CONCEA, Brazil) and approved by the Institutional Animal Care and Use Committee (CEUA), from Federal University of Rio de Janeiro (protocol no. 014/16; CEUA-UFRJ, Rio de Janeiro, Brazil).

### Virus Quantification

Mayaro virus titer and viral load in tissue samples were determined by plaque assay in BHK-21. Tissue samples were homogenized in α-MEM using a fixed relation of mass/volume and a serial dilution was prepared in α-MEM (10-fold). Then, each dilution was used to infect confluent BHK-21 cells seeded in 24-well plates. After 1 h of adsorption, the medium was removed and 2 mL of 1% carboxymethylcellulose (w/v) (Sigma-Aldrich) in α-MEM with 2% fetal bovine serum (FBS, Invitrogen) were added and cells were incubated at 37°C. After 48 h, cells were fixed using 4% of formaldehyde and plaques were then visualized by staining with 1% crystal violet in 20% ethanol. A title was calculated as plaque forming units per ml (pfu/ml) and converted to pfu/g.

### Histology

The gastrocnemius muscle and footpad were collected at defined days post infection and fixed with 4% of formaldehyde for 24 h. The footpad was decalcified using EDTA solution (125 g/L, pH 7.0) and then fixed. Tissues were embedded in paraffin after dehydration. Paraffin-embedded tissue sections of 5 μm were prepared and stained with hematoxylin and eosin (H&E). Images were obtained using optical microscopy with a magnification of 10 X (Olympus BX40), and images were acquired using software Leica Application Suite 3.8 (Leica).

### Cytokine Quantification by qPCR

Hind limb muscles were homogenized in DMEM using a fixed relation of 0.2 mg of tissue/μl, and 200 μl of the homogenate was used for RNA extraction with Trizol (Invitrogen) according to the manufacturer’s instructions. Purity and integrity of RNA were determined by the 260/280 and 260/230 nm absorbance ratios. One microgram of isolated RNA was submitted to DNAse treatment (Ambion, Thermo Fisher Scientific Inc) and then reverse-transcribed using the High-Capacity cDNA Reverse Transcription Kit (Thermo Fisher Scientific Inc). Quantification of cytokines expression was performed using the Power SYBR kit (Applied Biosystems; Foster City, CA, United States). Actin was used as an endogenous control. Primer sequences were described in Table 1.

**TABLE 1:** Primer sequences used for cytokines quantification by qPCR.

**Primer**	**Forward sequence**	**Reverse sequence**
ACTIN	TGTGACGTTGACATCCGTAAA	GTACTTGCGCTCAGGAGGAG
IL-6	TTCTTGGGACTGATGCTGGTG	CAGAATTGCCATTGCACACTC
IL-10	TAAGGGTTACTTGGGTTGCCAAG	CAAATGCTCCTTGATTTCTGGGC
IL-1β	GTAATGAAAGACGGCACACC	ATTAGAAACAGTCCAGCCCA
IFN-β	CCACTTGAAGAGCTATTACTG	AATGATGAGAAAGTTCCTGAAG
KC	CACCTCAAGAACATCCAGAGC	AGGTGCCATGAGAGCAGTCT
MCP-1	GTCCCCAGCTCAAGGAGTAT	CCTACTTCTTCTCTGGGTTG
RANTES	GTGCCCACGTCAAGGAGTAT	CCTACTTCTTCTCTGGGTTG
TGF-β	GACCGCAACAACGCCATCTA	AGCCCTGTATTCCGTCTCCTT
TNF	CCTCACACTCAGATCATCTTCTCA	TGGTTGTCTTTGAGATCCATGC

### Statistical Analyses

**FIGURE 1:**
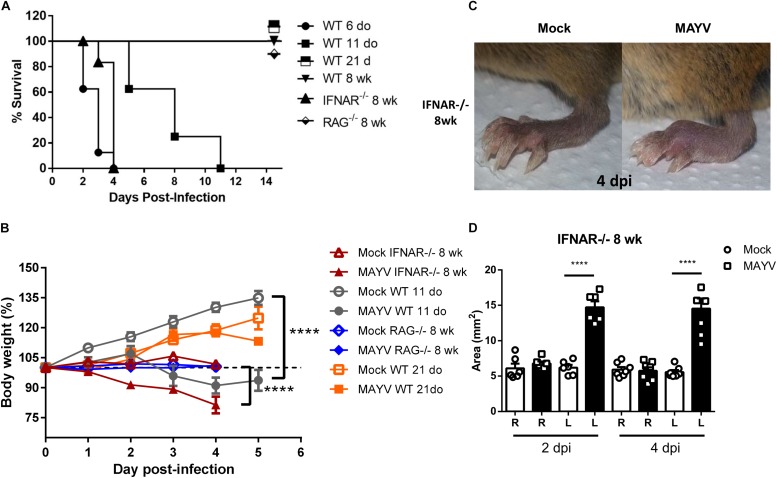
MAYV infection induces clinical signs in young WT and type I interferon receptor deficient mice. WT SV129 mice of different ages (do, days old), 8 week-old (wk) SVA129 (IFNAR^–/–^) and C57BL/6 RAG^–/–^ mice were subcutaneously infected with MAYV in the left footpad. **(A)** Survival was monitored up to 14 days post infection for mice in two independent experimental group of *n* = 5–6 mice each, and **(B)** body weight up to 5 days post infection of 11-day-old WT SV129 mice, Mock *n* = 7–9 and MAYV *n* = 6–10; 8-week-old IFNAR^–/–^, Mock *n* = 10 and MAYV *n* = 10; 8-week-old RAG^–^/^–^, Mock *n* = 5 and MAYV *n* = 10; 8-week-old WT SV129 mice, Mock *n* = 6 and MAYV *n* = 10, in each dpi. **(C)** Representative image of MAYV and Mock infected left footpad of 8 wk IFNAR^–/–^ mice. **(D)** The swelling area of left (L) and Right (R) paws of 8 wk IFNAR^–/–^ mice *n* = 6 was calculated using the measurement of paw height and width obtained by digital caliper at 2 and 4 days post infection. Values were plotted as mean ± Standard Error of Mean (SEM). Statistical analyses were performed by two-way ANOVA followed of Sidak’s multiple comparison test for weight gain curve (B) and one-way ANOVA followed of Tukey’s multiple comparison test for footpad swelling **(D)**. ^****^ < 0.0001.

We used two-way ANOVA followed of Sidak’s multiple comparison test to analyze the temporal weight gain curve at different groups (Figure 1B) and one-way ANOVA followed of Tukey’s multiple comparison test for the analyze MAYV-induced footpad swelling in IFNAR^–/–^ mice (Figure 1D). For analysis of MAYV tissue distribution and cytokines mRNA expression we used the non-parametric Mann-Whitney rank test. All tests performed using Graph Pad Prism version 7.00 for Windows, Graph Pad Software, La Jolla, CA, United States^1^.

## Results

### MAYV Inoculation Induces Clinical Signs of Infection in Young and Type-I Interferon Receptor Deficient Mice

Clinical findings suggested that MAYV induces a robust inflammatory response in patients, similar to other arthritogenic alphavirus (Santiago et al., 2015). However, MAYV tissue tropism and damage induced by infections has not been characterized yet. Hence, we first characterized whether MAYV infects or impacts animals in an age- and immunological status-dependent manner. For this, MAYV was inoculated in left hind limb footpad of young (6, 11, and 21 day-old) and adult WT SV129 mice. The infection resulted in high lethality and severe weight loss for young animals (≤11-days-old) (Figures 1A,B). Interestingly, 21-day-old infected-mice had no change in body weight when compared with Mock-infected mice and were completely resistant to MAYV-induced lethality, indicating their ability to control MAYV replication.

Inoculation of MAYV on adult WT SV129 and C57BL/6 mice did not cause any clinical signal of infection when compared to mock infected animals (Table 2 and Supplementary Figure S1A). However, inoculation of MAYV in type-I interferon receptor deficient (IFNAR^–/–^) adult mice results in early lethality and severe weight loss, similarly to infection of 6-day-old WT mice (Figures 1A,B). In addition, we observed intense paw edema in MAYV-injected footpad of IFNAR^–/–^ mice, when compared with mock (Figure 1C), or UV-inactivated MAYV infected footpad (data not shown). Measurement of edema area showed that the swelling in IFNAR^–/–^ was present at day 2 post infection (dpi) and maintained until 4 dpi (Figure 1D). No significant alteration was observed in the contralateral footpad and in the footpad of young and adult WT mice (Table 2). In addition to the swelling, IFNAR^–/–^ mice presented lethargy, locomotion dysfunction, and posterior weakness. Similar diversity and intensity of clinic signals were observed in 11-day-old WT mice (Table 2), differing only in the absence of swelling in the left paw.

**TABLE 2:** Clinical signs in MAYV infected mice.

	**Mice**						
**Clinical signs**	**IFNAR^–/–^ (SVA129) 8 wk**	**WT (SV129) 6 do**	**WT (SV129) 11 do**	**WT (SV129) 21 do**	**WT (SV129) 8 wk**	**WT (C56BL/6) 8 wk**	**RAG^–^/^–^ (C57BL/6) 8 wk**
Lethargy	+++	+++	+++	+	N	N	N
Weightloss	++	+	++++	+	N	N	N
Footpad swelling	++++	−	N	N	N	N	N
Locomotion dysfunction	+	−	++	N	N	N	N
Posterior weakness	++	++	++	N	N	N	N
Posterior paralysis	N	+	N	N	N	N	N

*Clinical signs were observed in different day-old (do) or week-old (wk) MAYV-infected mice. +, indicates presence and the amount of + the intensity of the signal. −, indicates that it was not possible to evaluate. N, absence of the signal.*

We have also investigated MAYV infection in adult recombination activation gene-1 deficient mice (RAG^–/–^), which preserved an active type-1 IFN response, but did not maturate B and T lymphocytes (Mombaerts et al., 1992), and used as a model to investigate the involvement of adaptive immunity in diseases (Haist et al., 2017). MAYV infection of adult RAG^–/–^ mice did not cause lethality, weight loss, and even other clinical signals observed in young WT and adult IFNAR^–/–^ mice (Table 2). Together, these results indicate that young mice and IFNAR^–/–^ are highly susceptible to MAYV infection, while adult mice are resistant even in the absence of lymphocytes.

### MAYV Replication Restriction Is Dependent on Innate and Adaptive Immunity

**FIGURE 2:**
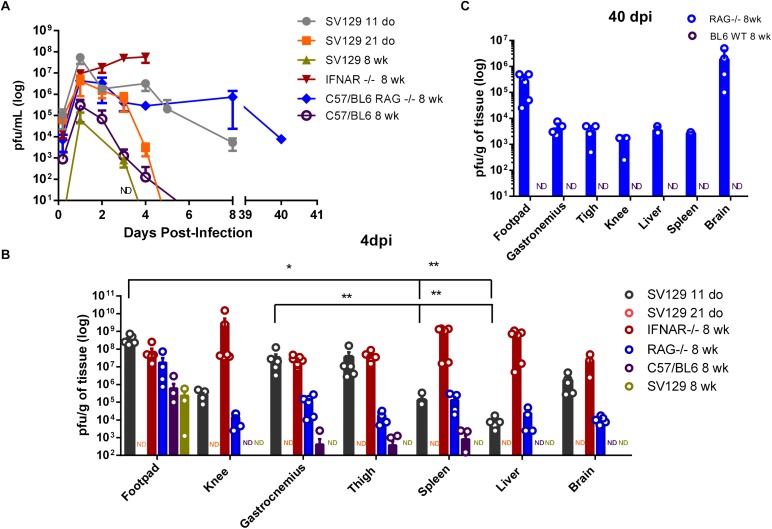
Control of viremia and tissue viral loads is dependent on age, innate, and adaptive immunity. WT SV129 mice of different ages (do, days old), 8 week-old (wk) SVA129 (IFNAR^–/–^) and C57BL/6 RAG^–/–^ mice were subcutaneously infected with MAYV in the left footpad. **(A)** Temporal analyses of MAYV infectious particles present in mice blood were determined by plaque assay with *n* = 4–6 mice per time-point at each group. **(B)** MAYV load at different tissues 4 days post infection. 11-day-old WT SV129 mice *n* = 4–6; 21-day-old WT SV129 *n* = 6; 8-week-old IFNAR^–/–^ *n* = 4–6; 8-week-old RAG^–^/^–^ *n* = 4–5 and 8-week-old WT C57BL/6 mice *n* = 6, as demonstrated by dot plots at figure. In some tissues the number of showed dots does not correspond to the n of experimental group due to the existence of animals with undetectable viral load. **(C)** 40 days post infection. 8-week-old RAG^–^/^–^ *n* = 3–5 and 8-week-old WT C57BL/6 mice *n* = 5, as demonstrated by dot plots at figure. Tissue samples were homogenized using a fixed relation of weight/volume and titled by plaque assay. Values were plotted as mean ± Standard Error of Mean (SEM). Samples without (no detection in any mice in the experimental group) in plaque assay was identified as ND. Statistical analysis on tissues within each group was performed using Mann-Whitney rank test. ^∗^ <0.05 and ^∗∗^ <0.01.

Subsequently, we performed a temporal analysis of MAYV viremia to correlate clinical signals with the ability to control viral burden. We observed an increase in the amount of MAYV infectious particles in the blood of mice of all ages on the first day of infection (Figure 2A). However, blood viral load was sustained at about 10^6^ pfu until 4 dpi in 11-day-old WT mice, while continuously decreased in 21 day-old WT mice until undetectable levels at 5 dpi, which reinforces the age-dependence for the control of MAYV infection. In agreement with this, the infection of WT SV129 and C57BL/6 adult mice results in a brief viremia. This ability to control viral replication was completely lost in the absence of type I interferon response, as observed by the continuous increase of viremia until the lethality of IFNAR^–/–^ mice (Figure 2A). Despite the absence of clinical signs and an intact INF response in adult RAG^–/–^ mice, MAYV infectious particle titers were sustained for a long period and maintained at about 10^4^–10^5^ pfu/ml until 7 dpi in the blood of RAG^–/–^ mice. Conversely, in this same period, MAYV was no longer detected in the blood of adult WT C57BL/6.

In order to identify preferential areas of replication, we evaluated the tissue distribution of MAYV at 4 dpi. MAYV infectious particles were detected in the articular and muscular tissues, as well as in liver, spleen, and brain of 11-day-old WT and adult IFNAR^–/–^ mice (Figure 2B). Although MAYV has a widespread distribution in mice tissues, we could observe a preferential distribution to the paw and the skeletal muscle in 11-day-old WT mice, since they presented a significantly higher viral load when compared to other peripheral tissue, as liver and spleen (Figure 2B). A high viral load was also detected in the footpad, knee, gastrocnemius and thigh muscle from the opposite side of the injection site in 11-day-old WT and adult IFNAR^–/–^ mice (Supplementary Figure S1B). These broad distributions were not observed in the infection of the 21-day-old and 8-week-old WT SV129 animals. MAYV was not detected in any tissue from 21-day-old mice and only detected in the footpad of adult SV129 mice at 4 dpi (Figure 2B and Supplementary Figure S1B).

MAYV was not detected in any tissue from 21-day-old mice and in the footpad of adult SV129 mice only at 4 dpi (Figure 2B and Supplementary Figure S1B). Infected adult WT C56/BL6 mice seem to be more permissive to MAYV replication than adult SV129, since still presenting low levels of MAYV 4 dpi in thigh, spleen and gastrocnemius (Figure 2B and Supplementary Figure S1B). However, MAYV was not detected in blood, liver, knee, or brain. These observations indicate that, similarly to SV129, adult C56/BL6 also controls MAYV replication and dissemination, even in a different time-course. In agreement with this, MAYV was not detected in 8 week-old C67/BL6 at 40 dpi (Figure 2C).

### MAYV Establishes Persistent Infection in the Absence of Adaptive Immunity

Although adult RAG^–/–^ mice presented a lower viral load in all tissues when compared to young WT and adult IFNAR^–/–^ mice 4 dpi, MAYV load was higher in RAG^–/–^ mice than in WT C57BL/6 mice and viral dissemination seems to be more efficient in RAG^–/–^ mice since it was detected in the brain tissue (Figure 2B). In addition, analysis of the blood and tissues of RAG^–/–^ mice demonstrated that MAYV continues to replicate actively in these animals until 40 days post infection, despite no apparent morbidity (Figures 2A,C). MAYV was detected in all analyzed tissues, but the highest viral load at 40 dpi was found in the left and right foot, and in the mice’s brains (Figure 2C and Supplementary Figure S1C). These results indicate that adaptive immunity is determinant for the elimination of MAYV, thus contributing in the avoidance of chronic infection.

### MAYV Induces Inflammation and Muscular Damage in Young WT and Adult IFNAR^–/–^ Mice

**FIGURE 3:**
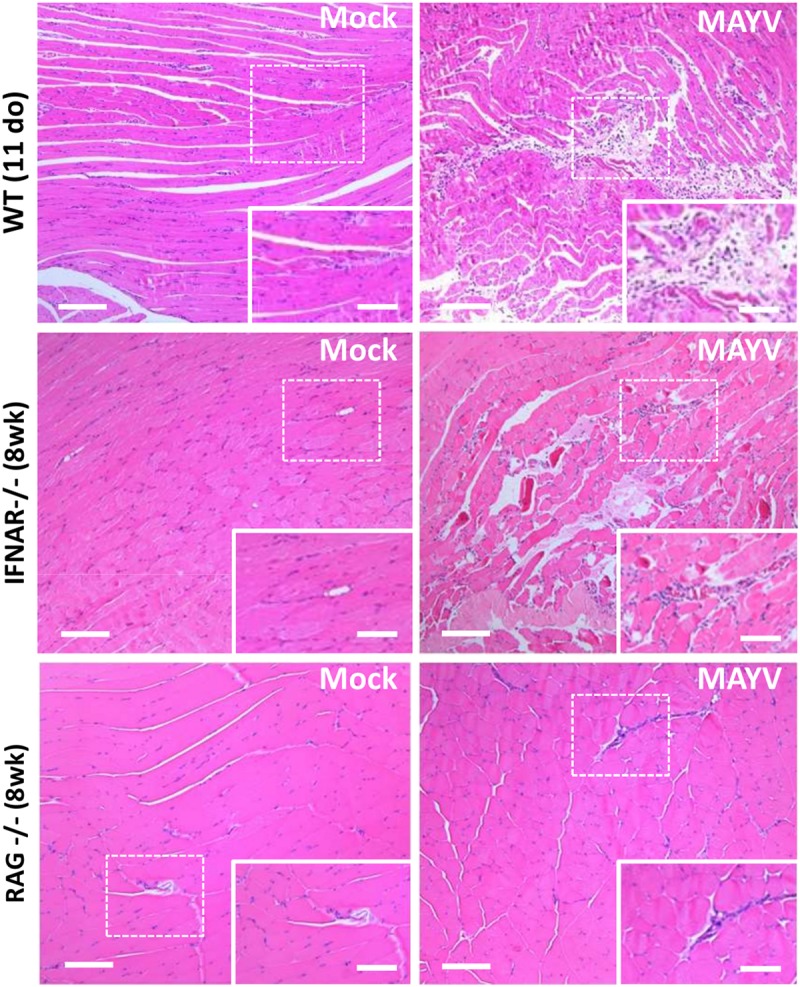
MAYV infection promotes muscle inflammation and necrosis in young WT and IFNAR^–/–^ mice. Eleven day-old (do) WT mice, 8 week-old (wk) IFNAR^–/–^, and RAG^–/–^ mice were subcutaneously infected with MAYV in the left footpad and gastrocnemius muscle tissue was collected and fixed at 4 dpi (WT and IFNAR^–/–^) or 40 dpi (RAG^–/–^) for histological analysis. Tissues were embedded in paraffin after dehydration and tissue sections of 5 μm were prepared and stained with H&E. Scale bar = 100 μm. Higher magnification images of the regions defined by dashed white rectangles (Scale bar = 10 μm).

Since joint and muscular tissues were the main sites of MAYV replication in 11-day-old mice, we investigated whether virus replication triggers pathological alterations. Histological analysis of H&E stained skeletal muscle of the hind limb of young WT and IFNAR^–/–^ mice showed sites of injury, with necrosis, edema, and infiltration of inflammatory cells at 4 dpi with MAYV (Figure 3). MAYV-induced muscular damage was similar in young WT and adult IFNAR^–/–^ mice. However, the extension of lesions seems to be higher in young WT mice. Despite the inability to clear the viral infection, no muscle damage was found in RAG^–/–^ mice in early infection (Supplementary Figure S2) and even at 40 dpi (Figure 3), correlating with the absence of clinical signals. This evidence indicates that adaptive immunity activation is determinant for viral clearance and could also be an important factor contributing to MAYV-induced inflammation and lesions.

**FIGURE 4:**
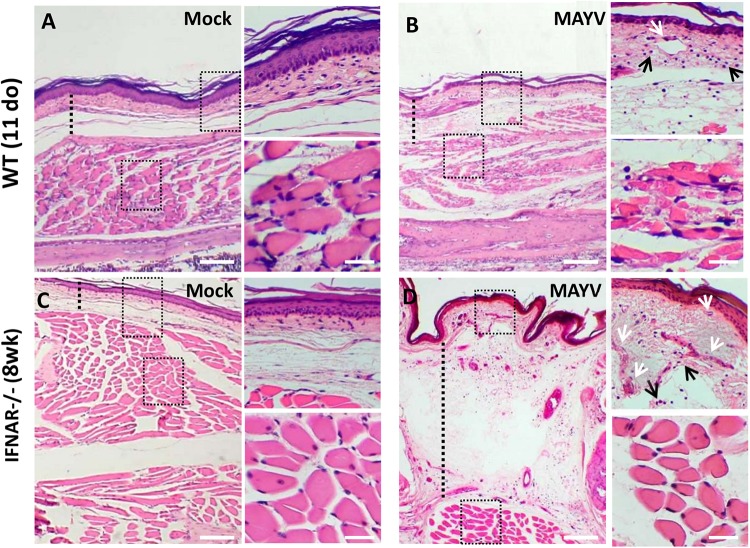
MAYV infection results in inflammation on the paw of young WT and IFNAR^–/–^ mice. 11-day-old (do) WT mice and 8-week-old (wk) IFNAR^–/–^ were subcutaneously infected with MAYV in the left footpad. Footpad longitudinal sections from MAYV- or Mock-infected animals (4 dpi) were stained with hematoxylin and eosin (H&E). Representative images of footpad from MAYV **(B,D)** – and Mock-infected animals **(A,C)**. As indicated by dashed line, MAYV-infected animals show footpad dermal edema (increased space between collagen), blood vessels congestion (white arrows), and inflammatory cell infiltration (black arrows). Scale bar of panoramic images = 500 μm. Higher magnification images of the regions defined by dashed black rectangles (Scale bar = 30 μm).

Analysis of H&E staining of the hind limb footpad at 4 dpi revealed that MAYV infection resulted in paw edema area which was close to articular-associated skeletal muscle (Figure 4), mainly in IFNAR^–/–^ infected mice and inflammatory cellular infiltration (Figures 4C,D). In addition, muscle damage was observed in young WT mice but not in IFNAR^–/–^, as can be observed in high magnification images (Figures 4A,B). Taken together, muscular and paw alterations indicate that MAYV replication in young and adult IFNAR^–/–^ mice results in damage and inflammation in target tissues. The damage induced by infection seems to correlate not only with the viral load, since higher loads of MAYV in the muscle and footpad of IFNAR^–/–^ do not results in higher damage, but might be triggered by inflammatory mediators and cellular activation during the course of infection.

### MAYV Infection Triggers a High Expression of Pro-inflammatory Mediators in Muscular Tissues

**FIGURE 5:**
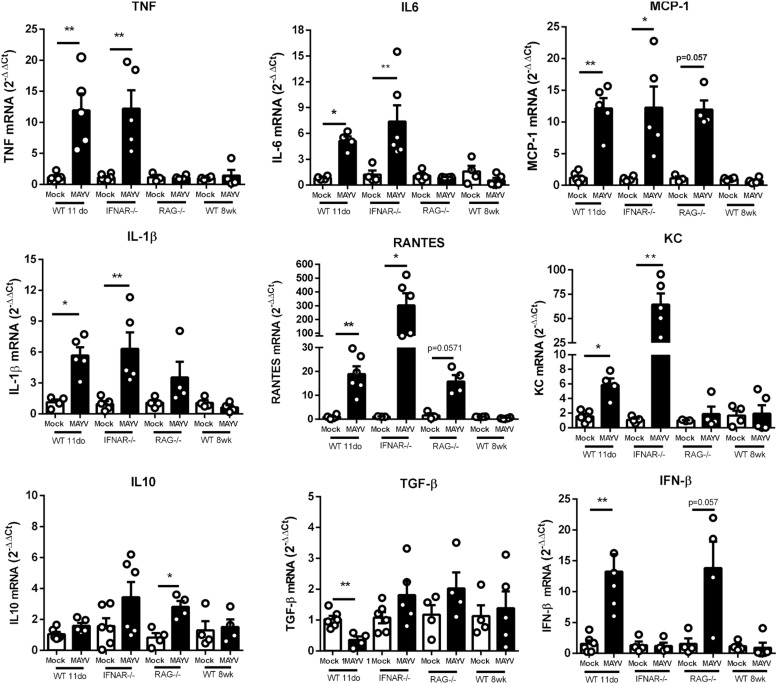
MAYV infection triggers a high expression of pro-inflammatory mediators in muscle tissue of young and IFNAR^–^/^–^ mice. 11-day-old WT SV129, 8-week-old IFNAR^–/–^, 8-week-old RAG^–^/^–^, and 8-week-old WT C57BL/6 mice were subcutaneously infected with MAYV in the left footpad. At 4 days post-infection, both posterior gastrocnemius muscles were collected and processed to determine the relative expression of pro-inflammatory cytokines. The levels of TNF-α, IL-6, MCP-1, IL-1β, RANTES, KC, IL-10, TGF-β, and IFN-β were determined through RT-PCR comparing gene expression with endogenous β-actin expression. The experimental group was: 11-day-old WT SV129 mice (Mock *n* = 4–5 and MAYV *n* = 4–5); 8-week-old IFNAR^–/–^ (Mock *n* = 4–6 and MAYV *n* = 4–6), 8-week-old RAG^–^/^–^ (Mock *n* = 3 and MAYV *n* = 4) and 8-week-old WT C57BL/6 mice (Mock *n* = 4 and MAYV *n* = 4), as demonstrated by dot plots at figure. Values are showed as mean ± Standard Error of Mean (SEM). Statistical analysis was performed using Mann-Whitney rank test. ^∗^*P* < 0.05 and ^∗∗^*P* < 0.01.

Some cytokines have been described as determinant to the progression of CHIKV induced lesions (Ng et al., 2009). Furthermore, MAYV patients with long-term articular symptoms present high concentrations of pro-inflammatory cytokines in their serum (Ng et al., 2009). Thus, we investigated if MAYV-induced damage was associated with the induction of a pro-inflammatory response in the muscular tissue of young WT, adult IFNAR^–/–^, and RAG^–/–^ mice. The quantification of inflammatory mediators expression by qPCR showed that MAYV replication in muscular tissues triggers high expression of cytokines and chemokines, such as TNF, IL-6, KC, IL-1β, MCP-1, and RANTES (Figure 5). The levels of cytokine induction following MAYV infection in IFNAR^–/–^ and young WT were very similar, except for RANTES and KC (Figure 5). Consistent with the histological observations in the muscle of adult RAG^–/–^ mice, the expression of some pro-inflammatory cytokines, such as TNF, IL-6, KC, and IL-1β, was not induced in early MAYV infection. However, we found a tendency of increased MCP-1 and RANTES levels (Figure 5). The alterations in muscle cytokines mRNA levels were not observed in adult C57BL/6 mice.

Since we found that the type-I interferon response was determinant for MAYV infection restriction, we also assessed whether MAYV was able to induce IFN-β expression in mice. We found that MAYV infection promotes an increase of about 15-fold of IFN-β mRNA expression in the muscle of young WT mice and a tendency of increase in RAG^–/–^ mice, but not in IFNAR^–/–^ mice (Figure 5). We also evaluated the expression of TGF-β and IL-10, important anti-inflammatory mediators related to a regenerative response. We found that TGF-β was down modulated in young WT mice, while being unaltered in adult IFNAR^–/–^ mice (Figure 5). IL-10 expression was slightly increased in RAG^–/–^, and not significantly altered in young WT and adult IFNAR^–/–^ mice infected with MAYV.

## Discussion

Host restriction of viral infection could be determined by several aspects of virus-cell interactions, including viral recognition and efficient innate and adaptive immune responses. Here we showed that MAYV replication induces clinical signals and muscular inflammation only in young WT and adult IFNAR^–/–^ mice. We found that MAYV presented a very narrow window of time to induce apparent infection in WT mice, with clinical signals been observed in 11-day-old mice but completely unapparent after 21 days of age. The clearance of viremia and absence of MAYV detection at 4 dpi in 21-day-old and adult mice reinforce that mice gain the capability to control virus dissemination with age. Viral replication in young mice could be associated with the inability of the immature immune system of mice to sustain an efficient antiviral response (Adkins et al., 2004; Zhang et al., 2017). This feature also correlates with clinical evidence of the severity and chronicity prevalence of Alphavirus infection in children and the elderly population (Lewthwaite et al., 2009; Hoarau et al., 2010; Murillo-Zamora et al., 2018).

The type I interferon (IFN-I) response has been described as essential to controlling RRV, CHIKV, ONNV, and SINV replication (Assuncao-Miranda et al., 2013). The induction of some interferon stimulated genes (ISG) is critical to restrict Alphavirus infection and dissemination (Brehin et al., 2009; Poddar et al., 2016; Carissimo et al., 2019). In agreement with this, adult IFNAR^–/–^ mice infected with MAYV develop a severe and lethal infection, indicating that IFN-I response is also important to restrict MAYV infection. However, we observed an increase of IFN-β expression in the muscle of infected young WT mice and also a tendency of induction in adult RAG^–/–^, which are both unable to control MAYV replication. It is possible that other factors could be associated with age-dependent severity of the infection or MAYV could inhibit IFNAR signaling in young.

Adaptive immunity seems to have a role in the controlling of MAYV infection, mainly in viral clearance. MAYV infection in RAG^–/–^ mice was completely unapparent and had no histological evidence of muscle damage, even with sustained viremia, and significant viral loads in tissues until 40 dpi. MAYV persisted mainly in joint-associated tissue and brain, but infectious particles were detected at lower levels in several peripheral tissues of the mice. The involvement of adaptive immunity in chronicity of CHIKV infection has been demonstrated, but its role in the process of tissue injury could be quite variable in different Alphavirus infection (Hawman et al., 2013; Seymour et al., 2015). As opposed to MAYV, CHIKV infected RAG^–/–^ mice presented chronic synovitis and myositis (Hawman et al., 2013; Seymour et al., 2015). Moreover, RRV induces muscle inflammation and disease in RAG^–/–^ mice during acute infection (Morrison et al., 2006). These data indicate that, despite the common involvement in arthritogenic Alphavirus persistence, the role of adaptive immunity in MAYV pathogenesis in mice could differ from this group, being an important issue that needs further investigation.

MAYV presented a broad tissue distribution in susceptible mice although in young WT mice the highest load was detected at muscular and articular tissues. In addition, we observed skeletal muscle necrosis and inflammation, mainly in young WT infected mice. The muscular tropism and damage could correlate with the main clinical symptoms observed in MAYV and is characteristic of myositis induced by other arthritogenic Alphavirus (Tesh et al., 1999; Ng, 2017). Currently, few studies investigated cellular tropism and consequences of MAYV replication (Cavalheiro et al., 2016; Camini et al., 2017; Caldas et al., 2018; Danillo Lucas Alves and Benedito Antonio Lopes da, 2018). It was demonstrated that MAYV replicates in RAW macrophages *in vitro*, promoting an increase in the amounts of reactive oxygen species and TNF (Cavalheiro et al., 2016). However, their roles in MAYV pathogenesis are not clear yet. Macrophage activation has a crucial role in articular and muscular inflammation-derived lesions in RRV and CHIKV infection (Lidbury et al., 2008; Gardner et al., 2010) and synovial macrophages have been described as viral persistence sites in infected patients with long-lasting arthralgia (Soden et al., 2000; Hoarau et al., 2010). In addition, the production of type I IFN by activated inflammatory monocytes has been suggested as crucial to control acute RRV infection in mice (Haist et al., 2017), but high levels of IFN-alpha mRNA were detected in the acute phase and in circulating monocytes of chronic CHIKV patients, indicating that virus persistence can occur despite antiviral immune activation (Hoarau et al., 2010; Wauquier et al., 2011). The presence of activated T cells was also detected in inflamed synovial and muscle tissue of CHIKV chronic patients (Ozden et al., 2007; Hoarau et al., 2010). Despite of the well-defined role of the inflammatory cellular infiltrate in tissue damage induced by CHIKV and RRV, the mechanism involved in the persistence of immune activation, and consequently the symptoms, is still not completely understood. Here, we observed moderate cellular infiltrate on the focus of necrosis and high expression of inflammatory mediators in the muscle of MAYV infected mice, including TNF and IL-6 mRNA levels that may contribute to tissue damage. Thus, it is possible that cells from the infiltrate could be responsible for the pro-inflammatory response in the muscle tissue.

Some studies evaluated cytokine profiles correlating with severity and chronicity of CHIKV infection (Ng et al., 2009; Hoarau et al., 2010; Chow et al., 2011; Kelvin et al., 2011). The severity of CHIKV was associated with the levels of IL1-β, IL-6, and RANTES (Ng et al., 2009), with IL-6 already associated with the persistence of symptoms (Chow et al., 2011). A similar study in MAYV infected patients was conducted by Santiago et al. (2015), showing high levels of TNF, IL-6, IL-8, IL-1Ra, IFN-γ, and others in the serum until 12 months post-acute phase (Santiago et al., 2015). Here we observed that acute MAYV infection in mice promotes elevated expression of inflammatory mediators in the muscle tissue, revealing a translation with MAYV induced disease in patients. In agreement with the involvement of pro-inflammatory cytokines in the severity of MAYV infection, we did not find tissue damage in RAG^–^/^–^ mice even after a long period of active replication. This observation could be determined by the lack of IL-6, TNF, and other inflammatory mediator’s induced as consequence of the lymphocyte mediated response.

Although the cytokine profile was similar to that seen in CHIKV infection, we cannot rule out differences in the amplitude of the inflammatory response. Interestingly, we observed that levels of TGF-β expression were reduced in young WT mice infected with MAYV. High levels of TGF-β were associated with a reduced immune response, persistent viremia, and with joint pathology in CHIKV infection in old mice (Uhrlaub et al., 2016). Therefore, the lower levels of TGF-β in MAYV infection could be important to sustain an immune response that restricts muscle damage. We also found a slight increase of IL-10 mRNA level in MAYV infected RAG^–^/^–^ mice, which is a cytokine involved in macrophage-induced muscle regeneration (Deng et al., 2012). However, we only measured the levels of cytokine mRNA that, due the ability of alphavirus to regulate protein translation machinery (Fros and Pijlman, 2016), could not reflect the protein levels synthesized. Further studies assessing the role of pro- and anti-inflammatory cytokines in the generation or resolution of muscle lesions are important for exploring the possibility of employing these mediators as new therapeutic targets.

Our study presents advances in the comprehension of molecular aspects involved in replication restriction of MAYV and tissue damage as a consequence of *in vivo* infection. The current knowledge about the mechanism of arthritogenic Alphavirus injury promotion was described using mainly CHIKV and RRV infection in mice models (Taylor et al., 2015; Haese et al., 2016). However, the characterization of similarities and specificities of Alphavirus-promoted diseases is important for the development of effective vaccines and therapies against this group of viruses. Thus, our study also represents an important contribution for further investigations on MAYV pathogenesis and also to test antiviral compounds and vaccines.

## Data Availability Statement

All datasets generated for this study are included in the manuscript/Supplementary Files.

## Ethics Statement

The animal study was reviewed and approved by all experimental procedures performed were in accordance with protocol and standards established by the National Council for Control of Animal Experimentation (CONCEA, Brazil) and approved by the Institutional Animal Care and Use Committee (CEUA), from the Federal University of Rio de Janeiro (Protocol No. 014/16; CEUA-UFRJ, Rio de Janeiro, Brazil).

## Author Contributions

IA-M, CMF, and RN conceived the experiments. CMF, RN, DG-L, MS, JA, and JD-S performed the experiments. IA-M, CMF, RN, MS, and CPF analyzed the results. IA-M wrote the manuscript. CPF, MB, and MTB reviewed and edited the manuscript.

## Conflict of Interest

The authors declare that the research was conducted in the absence of any commercial or financial relationships that could be construed as a potential conflict of interest.

## Footnotes

1 www.graphpad.com
