# Supplementary material for: Mayaro Virus Replication Restriction and Induction of Muscular Inflammation in Mice Are Dependent on Age, Type-I Interferon Response, and Adaptive Immunity
Source: Front Microbiol. 2019 Oct 1;10:2246. doi: 10.3389/fmicb.2019.02246 (PMC6779782; doi:10.3389/fmicb.2019.02246)
Supplement: Supplementary file 1 [file Data_Sheet_1.PDF]

Supplementary Figure 1

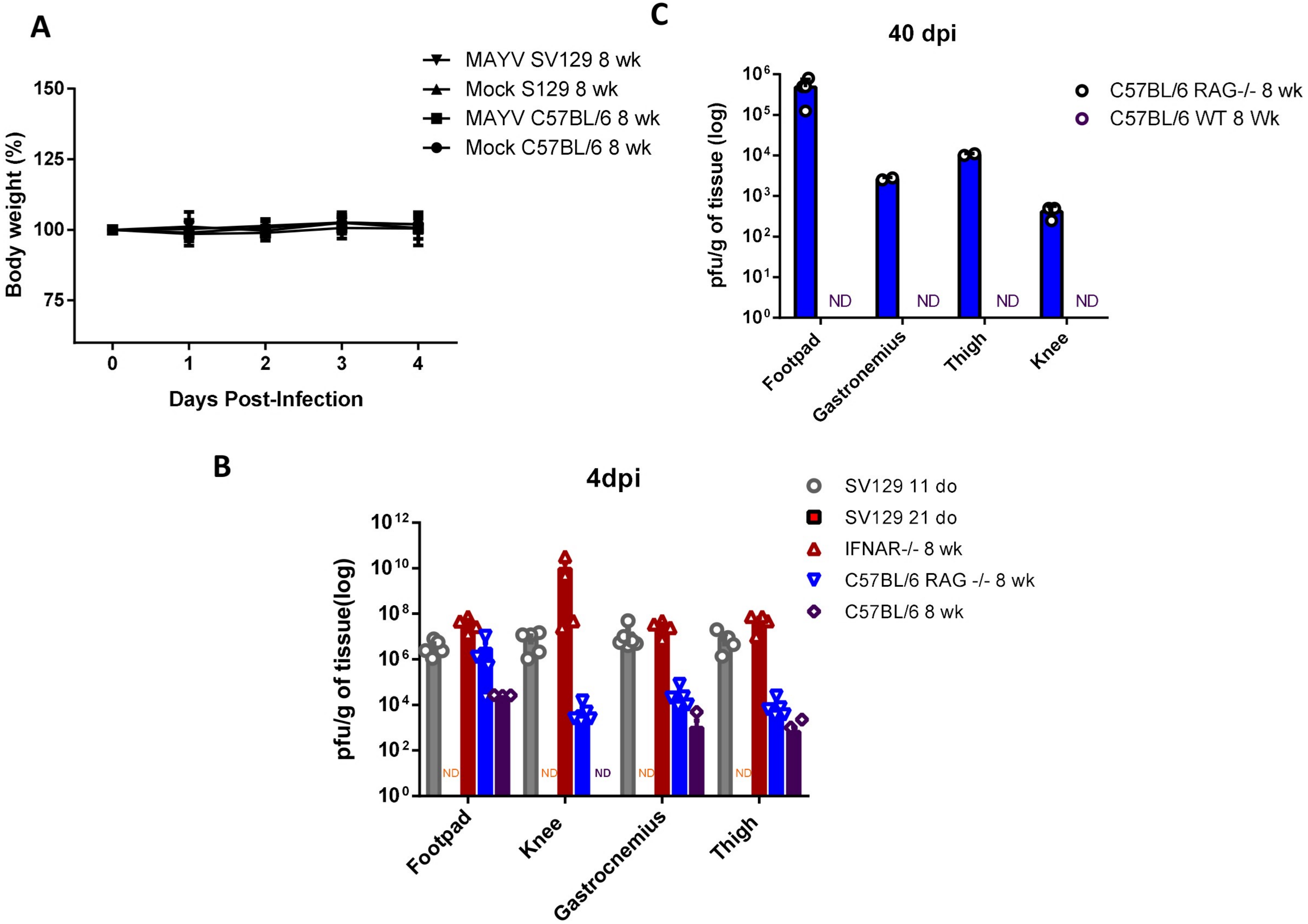

Supplementary Figure 1. (**A**) Body weight of Adult (8 weeks) WT SV129 mice (n=6) and C57BL/6 WT mice (n=6) was monitored throughout the days following the infection. Body weight was plotted in % using mass values in the moment of infection as reference. (**B**) MAYV load at right side muscular and articular tissues at 4 dpi of 11-day-old WT SV129 mice n=4-5; 21-day-old WT SV129 n=6; 8-week-old IFNAR$^{-/-}$ n=4; 8-week-old RAG-/- n=4-5 and 8-week-old WT C57BL/6 mice n=6, as demonstrated by dot plots at figure. **(C)** 40 dpi in 8-week-old RAG$^{-/-}$ (n=2-4) and 8-week-old WT C57BL/6 mice (n=5) right side tissue. Plot dots from 8-week-old WT C57BL/6 mice do not correspond to the n due to animals with undetectable viral load. Tissue samples were homogenized using a fixed relation of mass/volume and titled by plaque assay.

Supplementary Figure 2

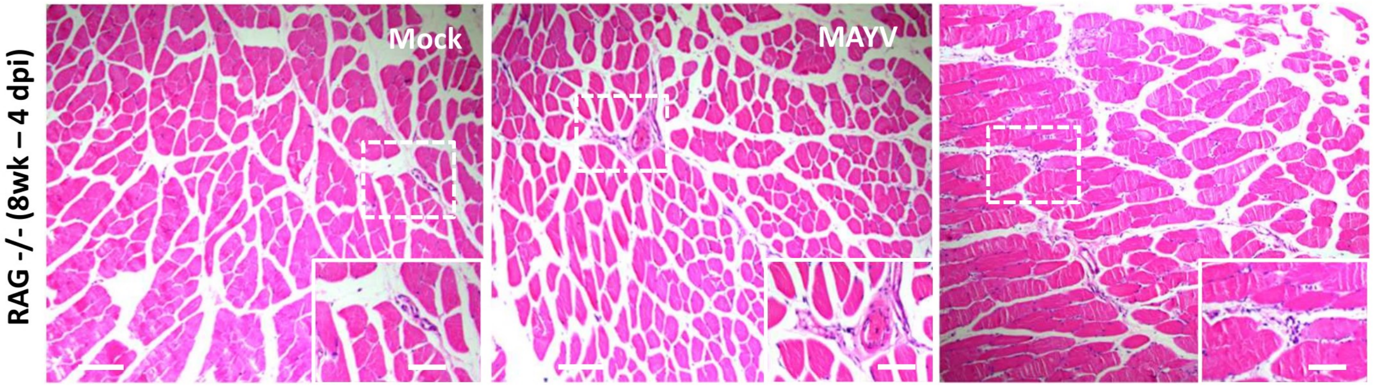

Supplementary Figure 2. MAYV infection do not results in acute muscle damage in RAG-/- mice. Eight-week old RAG1$^{-/-}$ mice were subcutaneously inoculated with MAYV or Mock in the left footpad and gastrocnemius muscle tissue was collected and fixed at 4 dpi for histological analysis. Tissues were embedded in paraffin after dehydration and tissue sections of 5 µm were prepared and stained with H&E. Scale bar = 100 µm. Higher magnification images of the regions defined by dashed white rectangles (Scale bar = 10 µm).
